# Impact of ARBs plus beta-blockers and other antihypertensives on cognitive impairment: a longitudinal study

**DOI:** 10.3389/fphar.2026.1954416

**Published:** 2026-10-21

**Authors:** Mar Garcia-Zamora, Gemma García-Lluch, Lucrecia Moreno, Juan Pardo, Consuelo Cháfer-Pericas

**Affiliations:** 1 Research Group in Alzheimer Disease. Instituto de Investigación Sanitaria La Fe, Valencia, Spain; 2 Cathedra DeCo MICOF-CEU UCH, Universidad Cardenal Herrera-CEU, CEU Universitites, Valencia, Spain; 3 Research Group in Neuroinmunology. Instituto de Investigación Sanitaria La Fe, Valencia, Spain; 4 Department of Pharmacy, Universidad Cardenal Herrera-CEU, CEU Universities, Valencia, Spain; 5 Department of Mathematics, Physics and Technology, Universidad Cardenal Herrera-CEU, CEU Universities, Valencia, Spain

**Keywords:** ACE inhibitors, antihypertensives, ARBs, beta-blockers, cognitive impairment, loop diuretics

## Abstract

**Background:**

Hypertension is a major modifiable risk factor for dementia, yet the specific neuroprotective benefits of different antihypertensive drug classes remain unclear. This longitudinal population-based cohort study evaluated the association between various antihypertensive agents, used as monotherapy or combination therapy, and the risk of cognitive impairment.

**Methods:**

The present work is a retrospective cohort study based on electronic health records (EHR) provided by the *Consellería de Sanitat Universal i Salut Pública* (Valencian Regional Health Authority, Spain) from two different hospitals in Valencia: Arnau de Villanova and Hospital La Fe. The study included 28057 individuals aged ≥65 years receiving antihypertensive therapy. Cox proportional hazards models, adjusted for demographic and clinical covariates, and Kaplan–Meier survival analyses were performed to estimate time-to-event outcomes and hazard ratios for cognitive impairment.

**Results:**

Loop diuretics and the combination of ARBs with beta-blockers, were associated with a reduced risk of cognitive impairment. Conversely, selective beta-blockers and angiotensin-converting enzyme (ACE) inhibitors were associated with an increased probability of impairment.

**Conclusion:**

Antihypertensive classes display divergent neuroprotective profiles. Loop diuretics, ARBs, and ARB–beta-blocker combinations appear protective, whereas ACE inhibitors and beta-blocker monotherapy show less favorable cognitive outcomes, highlighting the need for tailored pharmacological selection in aging populations.

## 1 Introduction

According to the World Health Organization (WHO), in 2021, the global prevalence of dementia was 57 million cases. Dementia is recognised as a public health priority (WHO, 2023). Although several new treatments have emerged in recent years (Claus et al., 2025), prevention remains crucial. It is estimated that 40% of dementia cases could be prevented or delayed by managing several risk factors (Santisteban et al., 2023). The Lancet Commission published a list of 14 modifiable risk factors for dementia (Livingston et al., 2024). Notably, cardiovascular diseases (CVD) such as diabetes, dyslipidaemia and hypertension (Santisteban et al., 2023; den Brok et al., 2021; Liampas et al., 2022; D’Silva et al., 2022; Holm et al., 2020) have been identified as significant risk factors for dementia (Livingston et al., 2024; Beaman et al., 2023; Nebel et al., 2018). Given that hypertension is a chronic condition requiring lifelong medication, it is imperative to comprehend the potential consequences of the various blood pressure-lowering drugs (Fan et al., 2024).

There are six classes of antihypertensive medications: alpha-adrenoreceptor antagonists, diuretics, beta-blockers, calcium channel blockers and drugs acting on the renin-angiotensin system (RAS), angiotensin-converting enzyme (ACE) inhibitors and angiotensin-receptor blockers (ARBs). Despite the divergent mechanisms through which these pharmaceutical agents act, they have been demonstrated to be efficacious in reducing blood pressure. Importantly, all antihypertensive drugs are small molecules and, therefore, potential candidates for passive diffusion (Carnovale et al., 2023).

Drug repurposing in neurodegenerative disorders such as Alzheimer’s disease (AD) offers the valuable opportunity to leverage existing clinical trial data on pharmacological effects, pharmacokinetics and safety profiles (Poblano et al., 2024; Morales et al., 2024). While several studies assessed that antihypertensives may have a beneficial effect on the incidence of dementia (den Brok et al., 2021; Liampas et al., 2022; Holm et al., 2020; Hughes et al., 2020), it remains unclear whether these benefits are due to better blood pressure control or to specific drug effects on dementia pathways. Crucially, rather than advocating for antihypertensive use in normotensive populations, evaluating these class-specific differences is essential to guide optimal pharmacological selection in patients who require treatment for hypertension. Ultimately, the question of which blood pressure-reducing agent offers superior protection against the development of all-cause dementia remains unanswered (den Brok et al., 2021; Liampas et al., 2022; Carnovale et al., 2023).

Furthermore, the management of hypertension often requires combination therapy to achieve optimal blood pressure control, especially in patients with moderate to severe hypertension or those with comorbid conditions. The rationale for combining antihypertensive drug classes is based on complementary mechanisms of action, mitigation of adverse effects, and evidence from clinical trials supporting efficacy and safety. Treatment should be individualized based on clinical profile, comorbidities, and response to therapy, with consideration of current guideline recommendations and trial evidence.

Consequently, there is a pressing need to understand the role of medications used to treat risk factors for dementia, such as antihypertensives. The present study aims to address this knowledge gap by evaluating the pharmacological effects of distinct antihypertensive classes, in monotherapy and combination, on the risk of cognitive impairment in older adults. Using a longitudinal time-to-event framework, we examined the time to cognitive impairment diagnosis while adjusting for key cardiometabolic covariates.

## 2 Materials and methods

### 2.1 Study design, setting and participants

The present work is a retrospective cohort study based on electronic health records (EHR) provided by the *Consellería de Sanitat Universal i Salut Pública* (Valencian Regional Health Authority, Spain), covering the period between 2002 and 2021. Data were obtained from two different hospitals in Valencia: Arnau de Villanova and Hospital La Fe.

The initial dataset was constructed from longitudinal clinical records comprising 233183 diagnostic instances of anonymized patients. Of the 2331833 individuals, we selected 28057 participants aged ≥65 years who received a first prescription for blood pressure-lowering drugs (Anatomical Therapeutic Chemical classification system (ATC) codes C02, C03, C07, C08, and C09) were included.

After the index date, participants could initiate additional cardiovascular therapies, including antidiabetics (A10), antithrombotic agents (B01), cardiac therapy drugs (C01), and/or lipid-lowering medications (C10).

To be eligible for inclusion, participants were required to have received antihypertensive treatment for a minimum period of 6 months This threshold was chosen to ensure a biologically plausible exposure period necessary for pharmacological influence on cognitive health, focusing on sustained adherence. For cognitively impaired individuals, the initiation of antihypertensive treatment had to occur at least 1 year before the clinical diagnosis. Medication use initiated after the clinical diagnosis was not considered in the analyses.

The index date was defined as the date of the first recorded antihypertensive prescription within the study observation period, marking the baseline entry into the study cohort. Because complete historical prescription data prior to study entry were unavailable to enforce a formal washout period, the cohort includes both incident and prevalent active users who met the inclusion criteria. A flowchart summarizing cohort selection and the study design, including the timing of medication exposure and outcome assessment, is presented in Figure 1.

**FIGURE 1:**
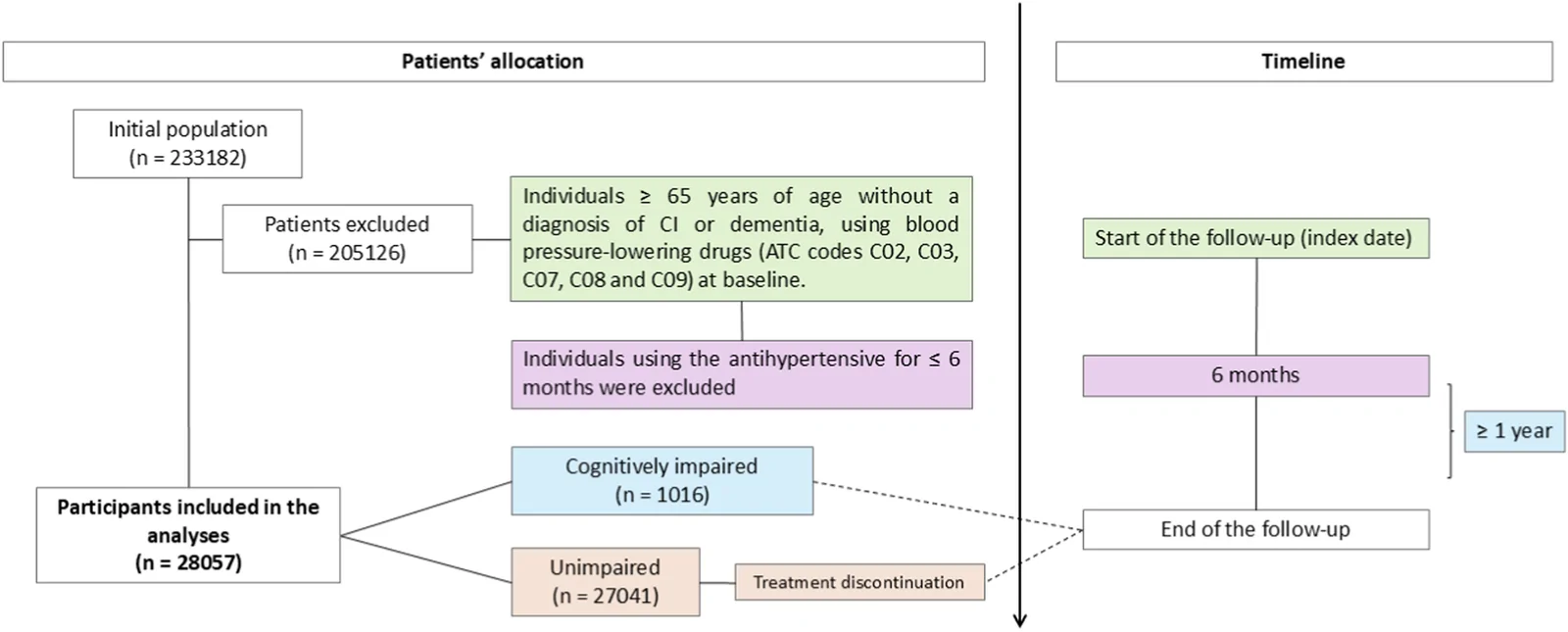
Patients’ allocation.

This research was conducted in accordance with the Declaration of Helsinki and approved by the Ethics Committee for Biomedical Research at CEU Cardenal Herrera University (CEEI23/423) and by the Ethics Committee at the Health Research Institute La Fe (CEIM22/537).

### 2.2 Drug exposure

Each blood pressure-lowering drug was first grouped into the second ATC level, encompassing antihypertensives (C02), diuretics (C03), beta-blocking agents (C07), calcium channel blockers (C08), and agents acting on the renin-angiotensin system (C09). Subsequently, the aforementioned groups were categorised within the fourth ATC level. Certain specific drug classes, including alpha-adrenoreceptor antagonists (C07AG), thiazides (C03AA), low ceiling sulphonamides (C03BA), aldosterone antagonists (C03DA), non-selective beta-blocking agents (C07AA), and alpha and beta-blocking agents (C07AG), which were not analysed due to the insufficient number of participants with a diagnosis of a neurodegenerative disorder using these drugs (n < 3).

To be considered exposed, participants had to have been taking the medication for at least 6 months. To avoid reverse causality and protopathic bias, a minimum 1-year lag period between treatment initiation and clinical diagnosis was required for impaired individuals.

Similarly, for participants who remained cognitively unimpaired during follow-up, exposure status was determined using the same treatment duration criteria of at least 6 months of continuous use starting from study entry. Follow-up ended at the earliest of the following: discontinuation of antihypertensive treatment, clinical classification as impaired, or end of study period.

Participants who discontinued treatment prior to receiving a diagnosis were censored at the exact date of discontinuation, and any diagnoses occurring after treatment cessation were not attributed to the exposed group.

Eligible blood pressure-lowering drugs were analysed in monotherapy and in combination. Monotherapy comprised participants who had only one antihypertensive drug class prescribed throughout the exposure window, whereas combination therapy included participants concurrently using ≥2 antihypertensive medications. Four participants who transitioned between monotherapy and combination regimens during the observation period were included in the overall cohort analyses but excluded from class-specific regimen subgroup analyses to maintain mutually exclusive treatment categories.

For each specific antihypertensive class evaluated in monotherapy, the exposed group consisted of users of that class, while the reference group comprised individuals using any other antihypertensive class in monotherapy. Similarly, for each specific combination therapy evaluated, the exposed group consisted of users of that specific combination, whereas the reference group comprised individuals receiving all other combination regimens.

### 2.3 Assessment of cognitive impairment

The diagnoses of MCI and AD were made using the International Classification of Diseases, Tenth Revision, Clinical Modification (ICD-10-CM codes) (World Health Organization, 1992), widely used in clinical data (Morales et al., 2024; Ding et al., 2025; Hsu et al., 2025; Soysal et al., 2019). Specifically, individuals with diagnostic codes beginning with G30 and G31 were classified as cognitively impaired individuals (n = 1016). In addition, those without these diagnosis codes were classified as cognitively unimpaired individuals (n = 27041).

Participants with any diagnosis of cognitive impairment before the index date were excluded. Furthermore, individuals diagnosed with other types of dementia (no AD), including mixed dementias were excluded from the analyses.

### 2.4 Covariates

Variables in the database can be classified as demographic or clinical. The demographic variables included age, sex, and health department. The clinical variables included the exposure time to the cardiovascular drug in days until the diagnosis or the end of the treatment in unimpaired individuals, and clinical diagnoses ascertained using the International Classification of Diseases, Tenth Revision, Clinical Modification (ICD-10-CM codes) and drug usage, which was determined through prescriptions using ATC codes: antidiabetic agents (A10), antithrombotic agents (B01), cardiac therapy drugs (C01), and lipid-lowering agents (C10).

### 2.5 Statistical methods

Descriptive analyses using Chi-square tests were carried out to compare the prescription of the different blood pressure-lowering drugs in monotherapy and in combination in cognitively impaired participants and cognitively unimpaired individuals. Furthermore, quantitative variables were analysed using the Student’s t-test.

Time-to-event analyses were performed using Cox proportional hazards models to estimate hazard ratios (HRs) and 95% confidence intervals (CIs) for the association between antihypertensive use (monotherapy and combination therapy) and becoming impaired. Two models were applied: Model 1, which was adjusted for sex and age; and Model 2, which was adjusted for sex, age, and the intake of antidiabetics, antithrombotic agents, cardiac therapy drugs and lipid-lowering agents.

Survival probabilities were estimated using unadjusted Kaplan–Meier curves, comparing incident users of each antihypertensive class in monotherapy with incident users of other antihypertensive regimens.

The proportional hazards assumption was tested using statistical methods. Schoenfeld residuals for each covariate and for the global model were calculated to identify non-proportionality. No significant violations were observed (all p-values >0.05).

Given the exploratory and hypothesis-generating nature of the study, no adjustment for multiple comparisons was applied (Bender and Lange, 2001; Rosendale et al., 2024). Consequently, conducting multiple statistical comparisons without multiplicity adjustment increases the probability of obtaining at least one false-positive finding. The reported p-values should therefore be regarded as nominal and interpreted descriptively and cautiously rather than as providing confirmatory evidence.

All statistical tests were two-sided. Nominal p-values <0.05 were used to identify potentially relevant associations for hypothesis generation. Analyses were performed using RStudio (version 2024.12.0 + 467) (Posi t team, 2024).

## 3 Results

### 3.1 Study population

Following the application of the inclusion and exclusion criteria, 28057 participants were selected (Figure 1). The mean follow-up was 1438 ± 879.3 days. Of these, 21167 were using blood pressure-lowering drugs in monotherapy. Among them, 848 (4.01%) were cognitively impaired. The remaining participants (n = 6886) were taking antihypertensive medications in combination, 6718 (97.56%) of whom were unimpaired, and 168 (2.44%) were classified as cognitively impaired.

### 3.2 Participants characteristics

Baseline characteristics are summarised in Table 1. Impaired participants were significantly older than unimpaired individuals [Unimpaired: 76.76 ± 7.90 vs. Impaired: 81.31 ± 6.63; p-value <0.001]. In addition, the percentage of women in this group was also significantly higher [Unimpaired: 15441 (57.10%) vs. Impaired: 651 (64.07%); p-value <0.001]. On the contrary, there were more men classified as cognitively unimpaired [Unimpaired: 11600 (42.90%) vs. Impaired: 365 (35.93%); p-value <0.001].

**TABLE 1:** Description of the general population, without making a distinction between monotherapy and combination.

Variables	Cognitively unimpaired (n = 27041)	Cognitively impaired (n = 1016)	*p* ^a^
Age, years	76.76 ± 7.90	81.31 ± 6.63	**<0.001**
Sex (n (%))			
Women	15441 (57.10%)	651 (64.07%)	**<0.001**
Men	11600 (42.90%)	365 (35.93%)	
Antidiabetics (A10), (n (%))	4726 (17.48%)	117 (11.52%)	**<0.001**
Antithrombotic agents (B01), (n (%))	7964 (29.45%)	239 (23.52%)	**<0.001**
Cardiac therapy (C01), (n (%))	1153 (4.26%)	28 (2.76%)	**0.023**
Blood pressure lowering drugs (C02, C03, C07, C08 and C09), (n (%))			
Alpha-adrenoreceptor antagonists (C02CA), (n (%))	1749 (6.47%)	67 (6.59%)	0.924
Loop diuretics (C03CA), (n (%))	7251 (26.81%)	233 (22.93%)	**0.007**
Beta blocking agents, selective (C07AB), (n (%))	6252 (23.12%)	239 (23.52%)	0.612
Dihydropyridine derivatives (C08CA), (n (%))	4874 (18.02%)	160 (15.75%)	0.068
ACE inhibitors (C09AA), (n (%))	3079 (11.39%)	125 (12.30%)	0.394
ARBs (C09CA), (n (%))	8774 (32.45%)	280 (27.56%)	**0.001**
Lipid modifying agents (C10), (n (%))	3795 (14.03%)	95 (9.35%)	**<0.001**

Test: Chi squared test and T-test.

Data are presented as mean ± standard deviation or number and proportion (%). Bold values indicate statistically significant differences

Abbreviations: ACE, angiotensin-converting enzyme; ARBs: angiotensin II receptor blockers p^a^ < 0.05.

According to the cardiovascular drugs, the percentage of people using antidiabetics [Unimpaired: 4726 (17.48%) vs. Impaired: 117 (11.52%); p-value <0.001], antithrombotic agents [Unimpaired: 7964 (29.45%) vs. Impaired: 239 (23.52%); p-value <0.001], cardiac therapy medications [Unimpaired: 1153 (4.26%) vs. Impaired: 28 (2.76%); p-value = 0.023] and lipid modifying drugs [Unimpaired: 3795 (14.03%) vs. Impaired: 95 (9.35%); p-value <0.001] was higher in the unimpaired group.

Finally, the intake of loop diuretics [Unimpaired: 7251 (26.81%) vs. Impaired: 233 (22.93%); p-value = 0.007] and ARBs [Unimpaired: 8774 (32.45%) vs. Impaired: 280 (27.56%); p-value = 0.001] was more prevalent among unimpaired individuals than in cognitively impaired participants.

### 3.3 Blood pressure-lowering drugs in monotherapy

Cox proportional hazards models (Table 2) indicated that the use of loop diuretics was associated with a decreased likelihood of being cognitively impaired in the two models studied (Model 1: [0.75 (0.62, 0.90); p-value = 0.002]; Model 2: [0.76 (0.63, 0.92); p-value = 0.005]).

**TABLE 2:** Hazard Ratios (HRs) and confidence intervals (CIs) for the risk of cognitive impairment according to antihypertensive monotherapy.

Monotherapy drugs	Model 1 [HR (CI 95%)]	*p* ^a^	Model 2 [HR (CI 95%)]	*p* ^a^
Alpha-adrenoreceptor antagonists (C02CA)	1.10 (0.79, 1.53)	0.570	1.08 (0.77, 1.50)	0.664
Loop diuretics (C03CA)	**0.75 (0.62, 0.90)**	**0.002**	**0.76 (0.63, 0.92)**	**0.005**
Beta blocking agents, selective (C07AB)	**1.18 (1.00, 1.39)**	**0.057**	**1.21 (1.02, 1.43)**	**0.026**
Dihydropyridine derivatives (C08CA)	1.02 (0.84, 1.25)	0.844	1.01 (0.83, 1.24)	0.900
ACE inhibitors (C09AA)	**1.35 (1.09, 1.66)**	**0.005**	**1.33 (1.08, 1.63)**	**0.008**
ARBs (C09CA)	0.91 (0.78, 1.06)	0.234	0.89 (0.76, 1.03)	0.121

Test: Cox proportional hazard regression.

Reference group: For each specific antihypertensive class, the reference group comprises participants receiving any other antihypertensive class in monotherapy.

Model 1: adjusted for sex and age.

Model 2: adjusted for sex, age, antidiabetics, antithrombotics, cardiac therapy drugs and lipid lowering agents. Bold values indicate statistically significant differences

Abbreviations: ACE, angiotensin-converting enzyme; ARBs: angiotensin II, receptor blockers *p* ^a^ < 0.05.

Among individuals using antihypertensives in monotherapy, the use of ACE inhibitors was more prevalent in cognitively impaired individuals (Supplementary Table S1) [Unimpaired: 1965 (9.67%) vs. Impaired: 102 (12.03%); p-value = 0.026], and their usage had the potential to increase the probability of becoming cognitively impaired (Table 2) (Model 1: [1.35 (1.09, 1.66); p-value = 0.005]; Model 2: [1.33 (1.08, 1.63); p-value = 0.008]). Additionally, the utilisation of selective beta-blocking agents significantly increased the likelihood of being impaired, although this effect was exclusively observed in Model 2 (Table 2) (Model 1: [1.18 (1.00, 1.39); p-value = 0.057]; Model 2: [1.21 (1.02, 1.43); p-value = 0.026]).

Finally, the intake of ARBs was the only factor that demonstrated statistical significance in the survival analyses. The utilisation of these antihypertensives resulted in higher survival probabilities to the event, i.e., participants who used ARBs had lower probabilities of becoming cognitively impaired (Figure 2).

**FIGURE 2:**
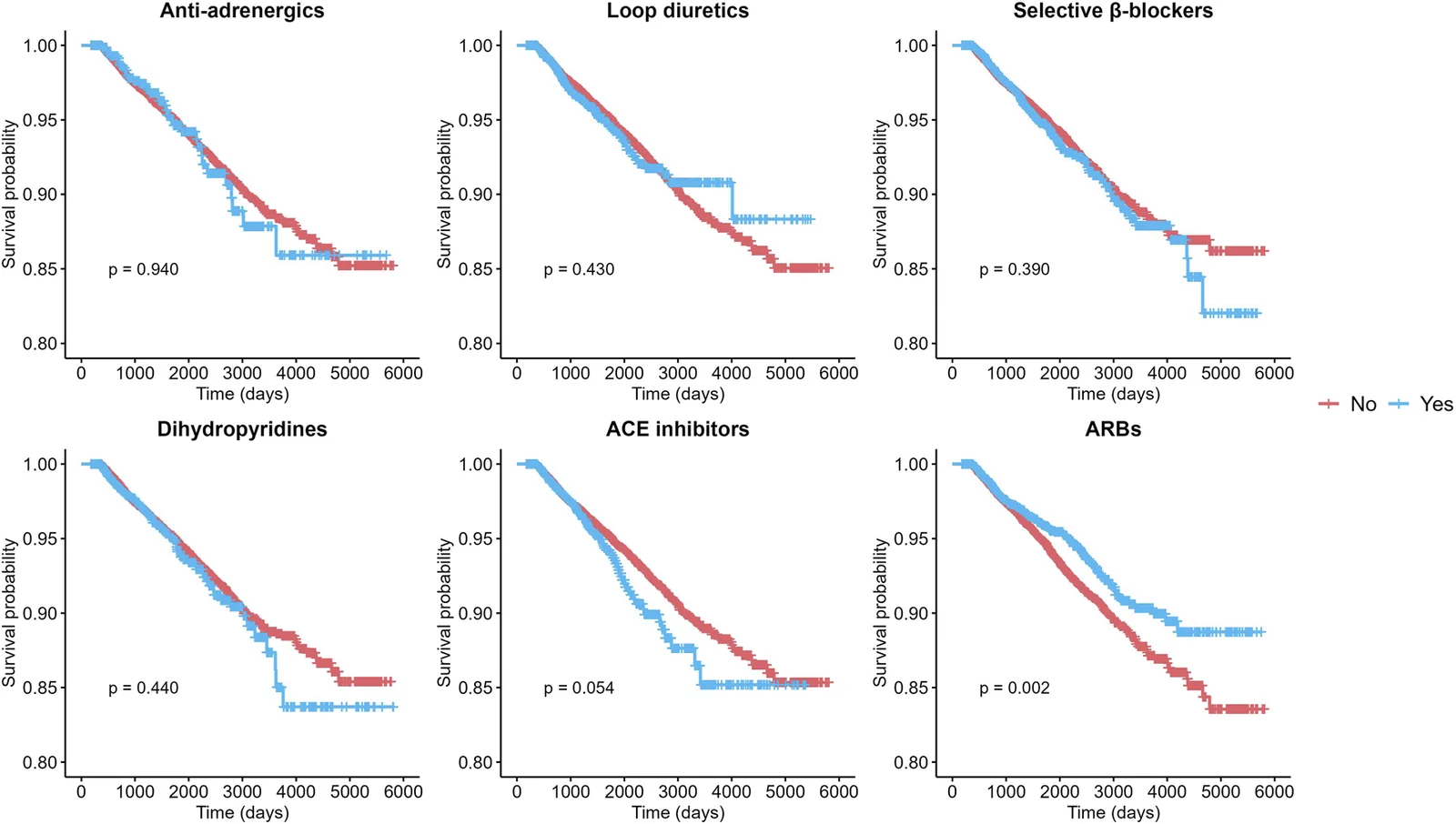
Kaplan Meier curves of the survival probability of developing neurodegenerative disorders in several antihypertensive medications*.* Blue stands for subjects using the blood pressure-lowering drug and red for those individuals not using it Abbreviations: ACE: angiotensin-converting enzyme; ARBs: angiotensin II receptor blockers.

### 3.4 Blood pressure-lowering drugs in combination

In individuals using a combination of antihypertensives, the intake of selective beta-blocking agents and ARBs was significantly more prevalent among cognitively unimpaired participants [Unimpaired: 867 (12.91%) vs. Impaired: 11 (6.55%); p-value = 0.015] (Supplementary Table S2). Furthermore, this same combination may decrease the likelihood of becoming cognitively impaired in both models of the study (Table 3) (Model 1: [0.52 (0.28, 0.96); p-value = 0.036]; Model 2: [0.54 (0.29, 0.99); p-value = 0.049]). The remaining combinations did not demonstrate statistical significance.

**TABLE 3:** Hazard ratios (HRs) and confidence intervals (CIs) for the risk of cognitive impairment according to antihypertensive combination.

Combination agents		Model 1 [HR (CI 95%)]	*p* ^a^	Model 2 [HR (CI 95%)]	*p* ^a^
Loop diuretics (C03CA) +	Beta blocking agents, selective (C07AB)	0.94 (0.63, 1.41)	0.769	1.07 (0.71, 1.61)	0.761
	Dihydropyridine derivatives (C08CA)	0.72 (0.42, 1.25)	0.250	0.75 (0.43, 1.30)	0.309
	ACE inhibitors (C09AA)	0.94 (0.51, 1.74)	0.850	1.01 (0.54, 1.86)	0.983
	Angiotensin II receptor blockers (ARBs) (C09CA)	0.76 (0.49, 1.18)	0.217	0.78 (0.50, 1.21)	0.272
Beta blocking agents, selective (C07AB) +	Dihydropyridine derivatives (C08CA)	1.11 (0.63, 1.96)	0.720	1.12 (0.64, 1.98)	0.693
	ACE inhibitors (C09AA)	1.04 (0.53, 2.03)	0.920	1.11 (0.56, 2.18)	0.762
	**ARBs (C09CA)**	**0.52 (0.28, 0.96)**	**0.036**	**0.54 (0.29, 0.99)**	**0.049**
Dihydropyridine derivatives (C08CA) +	ACE inhibitors (C09AA)	0.55 (0.20, 1.49)	0.242	0.58 (0.21, 1.56)	0.277
	ARBs (C09CA)	0.73 (0.42, 1.27)	0.264	0.72 (0.41, 1.24)	0.237

Test: Cox proportional hazard regression.

Reference group: For each specific combination therapy, the reference group comprises participants receiving all other antihypertensive combination regimens.

Model 1: adjusted for sex and age.

Model 2: adjusted for sex, age, antidiabetics, antithrombotics, cardiac therapy drugs and lipid lowering agents. Bold values indicate statistically significant differences

Abbreviations: ACE, angiotensin-converting enzyme; ARBs: angiotensin II, receptor blockers p^a^ < 0.05.

## 4 Discussion

In this large longitudinal study, conducted in 28057 individuals, we investigated the potential role of antihypertensive medications in cognitively impaired vs. unimpaired participants, yielding three key findings. First, the use of loop diuretics in monotherapy was associated with a 25% lower likelihood of being cognitively impaired. Second, selective beta-blockers and ACE inhibitors increased the likelihood of transitioning to cognitively impaired. Third, the combination of beta-blockers plus ARBs decreased by 45% approximately the likelihood of being classified as impaired.

Our results highlight the importance of drug selection in the management of hypertension, particularly in older adults. However, it is important to consider the context in which these drugs are prescribed. While hypertension is a well-established risk factor for cognitive decline (Livingston et al., 2024), effective pharmacological management may help preserve cognitive function. The magnitude of the inverse associations observed may suggest that these findings may reflect not only pharmacological effects but also underlying healthcare utilization and the management of concomitant medical conditions.

Our findings align with several studies that have shown the beneficial effects of diuretics (Sternberg et al., 2022; Peters et al., 2020) and ARBs (den Brok et al., 2021; Liampas et al., 2022; Peters et al., 2020; Wändell et al., 2019) on cognitive outcomes. In contrast, beta-blockers represent one of the five classes of drugs approved for the treatment of hypertension, particularly in patients with cardiovascular comorbidities such as heart failure (HF) (Taddei et al., 2024), atrial fibrillation and myocardial infarction (van Dalen et al., 2021). ACE inhibitors are also a suitable choice in both uncomplicated hypertension and in the context of coexisting coronary heart disease, HF (Santisteban et al., 2023) or diabetes mellitus (Santisteban et al., 2023; van Dalen et al., 2021). Consequently, the higher likelihood of impairment in these groups may be more attributable to the severity of the underlying medical conditions (confounding by indication) rather than a detrimental effect of the drugs themselves.

Interestingly, we observed a possible protective effect for the combination of ARBs plus beta-blockers suggesting a potential synergistic effect. This observation could be attributed to superior blood pressure management, as a recent meta-analysis demonstrated that combining beta-blockers with other antihypertensives, such as ARBs, achieves greater reductions in systolic and diastolic blood pressure (Guo et al., 2023). Alternatively, this combination may exert benefits beyond blood pressure control, supported by a case-control study showing that concomitant use of ARBs plus beta-blockers significantly reduced AD risk compared with statin users (Wang et al., 2023).

Indeed, the hypothesised mechanisms by which antihypertensives may prevent dementia focus on two broad areas: the effect of blood pressure itself and the specific neuroprotective effects of certain drug classes. Several meta-analyses have demonstrated that antihypertensive treatment reduces the risk of dementia through blood pressure reduction, regardless of the specific pharmaceutical agent employed (Belessiotis-Richards et al., 2025; Ding et al., 2020; Peters et al., 2022). Although our data cannot separate whether our findings are due to better blood pressure control or direct drug actions, we hypothesize that both mechanisms work together. Beyond lowering blood pressure, additional drug effects, such as reducing neuroinflammation, lowering oxidative stress, and improving brain blood flow, may offer independent protection. As shown in Figure 3, these combined mechanisms help explain the results observed in our study.

**FIGURE 3:**
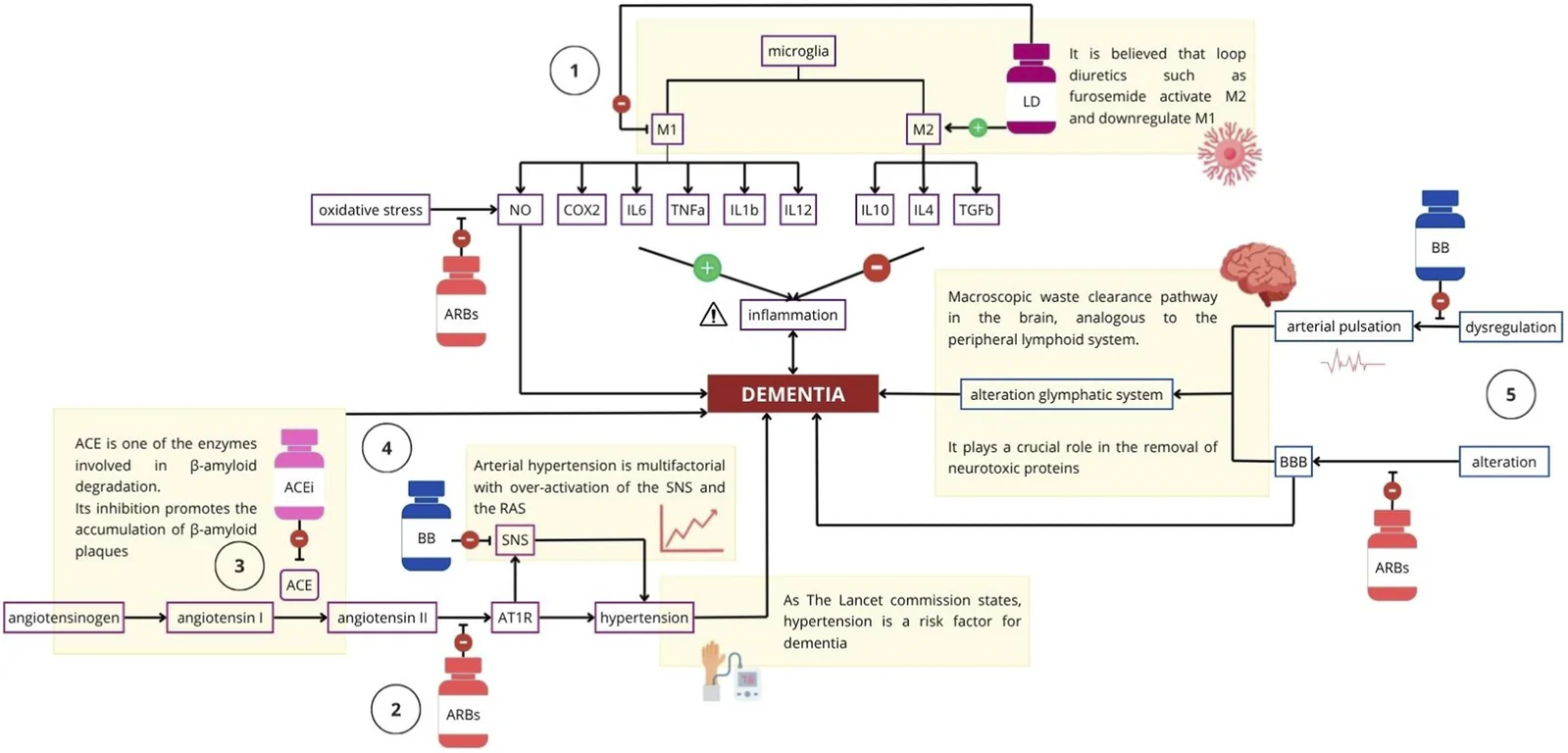
Simplified mechanism of action of the antihypertensive medications analysed in the study. Abbreviations: ACE: angiotensin-converting enzyme; ACEi: angiotensin-converting enzyme inhibitors; ARBs: angiotensin II receptor blockers; AT1R: angiotensin 1 receptor; BB: beta-blockers; BBB: brain blood barrier; LD: loop diuretics; SNS: sympathetic nervous system.

For instance, loop diuretics have been suggested to influence neuroinflammatory processes (Wang et al., 2021; Wang et al., 2020) and β-amyloid accumulation (Poblano et al., 2024), with specific agents like bumetanide currently under investigation as potential candidates for drug repurposing in AD (Cummings et al., 2025). Additionally, ARBs may promote neuronal survival and cerebral vasodilation through the modulation of angiotensin receptors (Liampas et al., 2022; Scotti et al., 2021; García-Lluch et al., 2023). Furthermore, the combination of ARBs and beta-blockers might target both the renin-angiotensin (D’Silva et al., 2022; Lebouvier et al., 2020) and the sympathetic nervous systems (do Vale et al., 2019; Lauren, 2017), potentially enhancing waste clearance in the brain through improved glymphatic flow.

Finally, regarding the clinical context, these findings must be interpreted within the framework of healthcare utilization and comorbidity management. In clinical practice, patients receiving complex or combination therapies often undergo more intensive monitoring by healthcare providers (Spanish Ministry of Health, 2022). This closer clinical follow-up may result in optimal management of chronic comorbidities, which could, in turn, contribute to better cognitive trajectories. Therefore, these associations may reflect a combination of pharmacological properties and the quality of overall medical care.

### 4.1 Strengths and limitations

The main strengths of this study are its large sample size, longitudinal design, and the use of ICD-10-CM codes for diagnosing neurodegenerative disorders.

However, several limitations should be acknowledged. First, drug exposure was inferred from prescriptions, without information on adherence. Second, although multivariable models were adjusted for demographic factors and key cardiometabolic co-medications (antidiabetics, antithrombotics, cardiac therapy, and lipid-lowering agents) as proxies for comorbidity burden, detailed data regarding baseline clinical parameters (such as blood pressure levels), lifestyle habits, and socioeconomic factors were unavailable, which may leave room for residual confounding. Third, follow-up did not include mortality data, potentially introducing bias. Fourth, because underlying clinical diagnoses driving prescriptions were not directly captured, associations with individual drug classes should be interpreted as descriptive and hypothesis-generating rather than causal. Furthermore, because no adjustment for multiple comparisons was performed, the probability of obtaining at least one false-positive association is increased. Therefore, nominally significant findings should be interpreted cautiously as descriptive and hypothesis-generating and require confirmation in independent cohorts or appropriately designed confirmatory studies. Fifth, the requirement of at least 6 months of continuous exposure and a 1-year lag period prior to diagnosis aimed to minimize reverse causality but may carry a risk of selection or immortal time bias. Additionally, because historical prescription data prior to cohort entry were incomplete and no washout period was applied, the index date represents operational entry into the study cohort rather than definitive first-ever medication use; thus, the inclusion of prevalent users may introduce prevalent-user bias and survivor bias. Finally, potential surveillance bias cannot be fully excluded, as individuals under chronic pharmacological monitoring are likely to experience more frequent medical encounters, increasing the probability of opportunistic cognitive impairment screening compared to unmonitored individuals.

Despite these limitations, longitudinal studies of this nature have the capacity to furnish valuable insights into the relationship between treatment usage and AD outcomes, thereby establishing new hypotheses for future studies.

## 5 Conclusion

The efficacy of different antihypertensive classes in the prevention of cognitive impairment is still a subject of debate. In this longitudinal population-based study, the combination of ARBs and beta-blockers reduced the likelihood of being cognitively impaired. The use of loop diuretics and ARBs also reduced this likelihood. In contrast, ACE inhibitors and beta-blockers used in monotherapy increased the likelihood of becoming cognitively impaired.

Antihypertensives may exert neuroprotective effects not only through blood pressure reduction but also by modulating neuroinflammation, oxidative stress, and cerebral perfusion.

However, given the observational nature of this study, these results should be interpreted with caution. The associations may be influenced by underlying patient characteristics, healthcare utilization patterns, or confounding by indication, particularly for drugs typically prescribed for more severe cardiovascular conditions. Therefore, this research should be regarded as hypothesis-generating rather than definitive evidence of causality. Our findings serve as a starting point for better-targeted prospective studies designed to further explore the complex relationship between antihypertensive selection and cognitive decline in older adults.

## Data Availability

The data that support the findings of this study are available from the Health Authority (Conselleria de Sanitat) upon reasonable request and subject to formal data access authorization and ethics committee approval. Restrictions apply to the availability of these data, which were used under license for the current study, and so are not publicly available or directly transferable by the authors.
