# Supplementary material for: Impact of ARBs plus beta-blockers and other antihypertensives on cognitive impairment: a longitudinal study
Source: Front Pharmacol. 2026 Oct 21;17:1954416. doi: 10.3389/fphar.2026.1954416 (PMC13731390; doi:10.3389/fphar.2026.1954416)
Supplement: Supplementary file 1 [file Supplementaryfile1.docx]

**Supplementary Table 1.** Descriptive analyses of the different blood pressure-lowering drugs in monotherapy in unimpaired individuals vs cognitively impaired

| **Variables (21167)** | **Cognitively unimpaired**  **(n = 20319)** | **Cognitively**  **impaired**  **(n = 848)** | ***p*^a^** |
| --- | --- | --- | --- |
| Alpha-adrenoreceptor antagonists (C02CA), (n (%)) | 766 (3.77%) | 37 (4.36%) | 0.380 |
| Loop diuretics (C03CA), (n (%)) | 3847 (18.93%) | 139 (16.39%) | 0.062 |
| Beta blocking agents, selective (C07AB), (n (%)) | 3683 (18.13%) | 175 (20.64%) | 0.073 |
| Dihydropyridine derivatives (C08CA), (n (%)) | 2642 (13.00%) | 111 (13.09%) | 0.963 |
| **ACE inhibitors (C09AA), (n (%))** | **1965 (9.67%)** | **102 (12.03%)** | **0.026** |
| ARBs (C09CA), (n (%)) | 5983 (29.45%) | 227 (26.77%) | 0.117 |
| **Test:** Chi squared test  Data are presented as number (proportion %)  **Abbreviations**: ACE, angiotensin-converting enzyme; ARBs, angiotensin II receptor blockers  *p*^a^ < 0.05 | | | |

**Supplementary Table 2.** Descriptive analyses of the different blood pressure-lowering drugs in combination in unimpaired individuals vs cognitively impaired

| **Variables (6886)** | | **Cognitively unimpaired**  **(n = 6718)** | **Cognitively impaired**  **(n = 168)** | ***p*^a^** |
| --- | --- | --- | --- | --- |
| Loop diuretics (C03CA) + | Beta blocking agents, selective (C07AB), (n (%)) | 1126 (16.76%) | 28 (16.67%) | 0.975 |
|  | Dihydropyridine derivatives (C08CA), (n (%)) | 736 (10.96%) | 14 (8.33%) | 0.280 |
|  | ACE inhibitors (C09AA), (n (%)) | 410 (6.10%) | 11 (6.55%) | 0.810 |
|  | Angiotensin II receptor blockers (ARBs) (C09CA), (n (%)) | 1046 (15.57%) | 23 (13.69%) | 0.506 |
| Beta blocking agents, selective (C07AB) + | Dihydropyridine derivatives (C08CA), (n (%)) | 512 (7.62%) | 13 (7.74%) | 0.954 |
|  | ACE inhibitors (C09AA), (n (%)) | 369 (5.49%) | 9 (5.36%) | 0.942 |
|  | **ARBs (C09CA), (n (%))** | **867 (12.91%)** | **11 (6.55%)** | **0.015** |
| Dihydropyridine derivatives (C08CA) + | ACE inhibitors (C09AA), (n (%)) | 288 (4.29%) | 4 (2.38%) | 0.225 |
|  | ARBs (C09CA), (n (%)) | 811 (12.07%) | 14 (8.33%) | 0.140 |
| **Test:** Chi squared test  Data are presented as number (proportion %)  **Abbreviations**: ACE, angiotensin-converting enzyme; ARBs, angiotensin II receptor blockers  *p*^a^ < 0.05 | | | | |
